# Evaluation of serum immunoglobulin E levels in bronchial asthma

**DOI:** 10.4103/0970-2113.68312

**Published:** 2010

**Authors:** Thirunavukkarasu Sandeep, Mysore Subrahmanyam Roopakala, Chickballapur Rayappa Wilma Delphine Silvia, Srikantaiah Chandrashekara, Mohan Rao

**Affiliations:** *Department of Physiology, MS Ramaiah Medical College, Bangalore – 560054, India*; 1 *Department of Biochemistry, Vydehi Institute of Medical Sciences and Research Centre, Bangalore- 560066, India*; 2 *Department of Immunology and Rheumatology, MS Ramaiah Medical College, Bangalore – 560054, India*; 3 *Department of Chest Medicine, MS Ramaiah Medical College, Bangalore – 560054, India*

**Keywords:** Allergy, bronchial asthma, immunoglobulin E, inflammation

## Abstract

**Background:**

Immunoglobulin E and associated cellular responses are responsible for allergic airway diseases. A hypersensitivity reaction initiated by immunologic mechanisms, mediated by IgE antibodies occurs in allergic asthma

**Aim:**

To estimate and compare serum IgE levels in mild, moderate, and severe asthmatics and in normal subjects and to obtain a mathematical model describing the relationship between serum IgE levels and severity of asthma.

**Materials and Methods:**

A stratified sample of 60 patients within age group of 18-60 years and 31 male and 29 female asthmatic patients and 13 healthy controls within 18-60 years were included in this study and classified according to GINA classification. Serum IgE levels were estimated by using ELISA kit.

**Results:**

Mean IgE levels ranged from 151.95 IU/ml in normal subjects to 1045.32 IU/ml in severe asthmatics. The model developed was 27% efficient.

**Conclusion:**

Serum Immunoglobulin E levels were high in asthmatics as compared to normal subjects. On an average, the levels increased as the severity of asthma increased. However, there was no statistically significant correlation since the variability in each level of asthma was very large

## INTRODUCTION

Allergic respiratory disorders, in particular asthma, are increasing in prevalence, which is a global phenomenon. Although profound insights have been made into the pathophysiology of bronchial asthma, the exact mechanisms inducing and regulating the disease are still not fully understood. Even though genetic predisposition is one of the factors in children for the increased prevalence, urbanization, air pollution and environmental tobacco smoke contribute more significantly. Recent studies in the city of Bangalore show a prevalence of 29.5% in children below the age of 18 years.[1]

Allergic diseases including asthma are characterized by an increase of serum Immunoglobulin E (IgE) levels.[2,3] A hypersensitivity reaction initiated by immunologic mechanisms mediated by IgE antibodies occurs in allergic asthma. IgE plays a central role in the initiation and propagation of the inflammatory cascade and thus the allergic response.[4] Indeed, recent studies reveal that IgE, through its high affinity IgE receptors (Fc epsilon R1), is a critical regulator of T_H_2 responses.[2]

Since the time of its discovery in 1966, IgE has been considered the most important biological target in the treatment of allergy and asthma with many investigators trying to interfere with its production or its function in the immune system. This is supported by the success of the anti-IgE monoclonal antibody (mAb) in the treatment of allergy and asthma. Nonetheless, adverse reactions such as anaphylaxis, urticaria and serum sickness have been reported with this therapy and repeated injections at extremely high costs are required to maintain effectiveness.[2]

Hence, without separating asthma patients whose disease is largely dependent on the allergic response from those whose asthma also may result from other factors such as an anti-viral reaction, it may be impossible to discern the drug’s true effectiveness on allergy-triggered asthma. We undertook this study to estimate and compare serum IgE levels in mild, moderate, and severe asthmatics and in normal subjects and to obtain a mathematical model describing the relationship between serum IgE levels and severity of asthma.

## MATERIALS AND METHODS

A cross sectional sample of urban adult population in Bangalore was considered. The study was conducted at MS Ramaiah Medical Teaching Hospital, Bangalore. A detailed explanation of the purpose of the study, the procedure adopted and the safety measures undertaken while sampling blood were clearly given to the asthma patients attending the out patient department of chest medicine of the hospital. 31 male and 29 female patients in the age group of 18-60 years, with an acute attack of bronchial asthma, volunteered to participate in the project; 13 healthy volunteers within 18 -60 years as control group were enrolled after taking an informed consent from them. Asthmatics who had taken bronchodilators within 24 hours prior to assessment, other allergic disorders, immunocompromised patients, chronic respiratory diseases other than asthma were excluded from the study. The study was approved by the institution’s Ethical Committee, and informed consent was obtained from every subject.

For each of the patients the Forced Expiratory Volume 1 (FEV1) and IgE levels were measured. The pulmonary function test was done using computerized spirometry – Spirobank G. Severity of asthma was categorized as mild, moderate and severe based on GINA (Global Initiative for Asthma) guidelines.[5]

After taking the necessary aseptic precautions from the median cubital vein, two ml of venous blood was collected from each patient using vacutainers. After collecting the blood samples they were left undisturbed for about half an hour for complete clot formation. The sample was then centrifuged to separate the serum from the clot. After centrifugation the serum was stored at −20° C in eppendorf tubes till the analysis was done.

Serum IgE levels were measured using Genesis Diagnostics ELISA kit. It is a solid phase enzyme linked immunosorbent assay based on the sandwich technique. Organon Teknika Microwell system was used to estimate the concentration of IgE at 450 nm.

Statistical analysis was performed with Student’s t test by using SPSS version 10.0. A value of *P* < 0.05 was taken to indicate statistical significance.

## RESULTS

The mean Ig E levels in healthy controls and cases were found to be 151.95 IU/L and 756.26 IU/L respectively and statistically significant when the levels were compared between these two groups (*P* < 0.001).

In this study the asthmatics were categorized into three groups based on the GINA guidelines viz mild, moderate and severe group [Table 1]. The patients in each group had similar demographic characters. Their pulmonary function tests were significantly different among the three groups. This agrees with the classification criteria used – GINA guidelines. The mean FEV1 values in the mild, moderate and severe asthmatic groups were 67.77%, 62.82% and 42.42% respectively.

**Table 1:** Serum Ig E levels in controls and cases

Groups	Number of subjects	Mean (IU / ml)
Normal	13.00	151.95
Mild asthma	19.00	464.00
Moderate asthma	18.00	695.41
Severe asthma	23.00	1045.32

## DISCUSSION

Asthma is of two types - extrinsic and intrinsic types. Atopic asthma is a subtype of extrinsic asthma in which patients who have a hyper-responsive airway, the scene for the reaction is set in large part by initial sensitization to inhaled antigens and chemical antigens.[6,7] These stimulate induction of T_H2_ type T cells, which release cytokines like IL- 4 and IL- 5. The released cytokines in turn promote IgE production by B cells, growth of mast cells (IL- 4) and growth and activation of eosinophils. Subsequent IgE mediator reaction to inhaled allergens elicits acute and late phase reaction.[8]

The analysis of the data revealed that mean IgE levels were significantly different among the groups with *P* <0.001. It also indicated that there is trend in IgE levels – lowest in normal group and highest in severe group. This shows the important role played by IgE in the severity of asthma; but whether this high level is a causative factor in severity of symptoms could not be ascertained. The limitation of this study is that the status of the fungal sensitization in the patients specifically the work-up for allergic bronchopulmonary aspergillosis (ABPA) was not carried out in asthmatics especially those found to have IgE levels more than 1000 IU/mL

There are various other factors involved in the causation of bronchial asthma because other cells in affected bronchi can also produce the mediators; this is inclusive of vascular endothelium, airway epithelium and inflammatory cells already present, in asthmatics suffering a recurrent attack.[9] It may be proposed that the levels of IgE are quite high locally at the site of inflammation and the serum levels do not necessarily reflect the levels in lungs and bronchus. It is also known that IgE is bound to mast cells with rather high affinity[8] and hence the circulating IgE may not give a conclusive evidence of the severity of inflammation.

Increase in IgE may be due to viral infections, which is the commonest cause of exacerbation of symptoms in asthma or may simply represent a generalized up-regulation of IgE production.[10] Systemic IgE have been found to increase following infection with Epstein Barr virus (EBV),[11] Cytomegalo virus (CMV), Measles virus,[12] vaccination with whole virion influenza vaccine[13] and Rhinovirus.[14]

## CONCLUSION

Serum Immunoglobulin E levels were high in asthmatics as compared to normal subjects. On the average, the IgE levels increased as the severity of asthma increased. Hence it may be concluded that though the levels of IgE differ significantly with severity of asthma it does not completely explain the severity of the symptoms and signs. There are other factors like cytokines which are responsible for the clinical presentation. The very fact that the anti-IgE therapies developed are not 100% effective implies that IgE may not be the sole root cause for asthma.[15]
